# Effect of sequential coronary artery bypass venous grafting on right ventricular functions assessed by tissue Doppler echocardiography

**DOI:** 10.5830/CVJA-2010-093

**Published:** 2012-03

**Authors:** G Ozerdem, N Katrancioglu, O Berkan, B Candemir, E Saricam, O Ozturk

**Affiliations:** Department of Cardiovascular Surgery, Cag Hospital, Ankara, Turkey; Department of Cardiovascular Surgery, Cumhuriyet University School of Medicine, Sivas, Turkey; Department of Cardiovascular Surgery, Cumhuriyet University School of Medicine, Sivas, Turkey; Department of Cardiology, Ankara University School of Medicine, Ankara, Turkey; Department of Cardiology, Cag Hospital, Ankara, Turkey; Department of Cardiology, Cag Hospital, Ankara, Turkey

**Keywords:** sequential bypass grafting, right ventricle, echocardiography, diastolic function, coronary artery disease

## Abstract

**Background:**

Coronary artery bypass graft surgery is a well-known and proven method of treatment for coronary artery disease. A modification of this method is complete revascularisation of the right ventricle by sequential bypass grafting of the right coronary artery, the effects of which on ventricular function need to be clarified. We sought to determine the effect of the sequential bypass graft method on right ventricular (RV) function utilising tissue Doppler echocardiography.

**Methods:**

A total of 35 coronary artery disease patients (group A: 20 sequential grafts; group B: 15 individual grafts) were enrolled. Patients were examined pre-operatively with tissue Doppler echocardiography for RV function, and again postoperatively after the first month.

**Results:**

Pre-operatively, there were no significant differences with regard to demographics or basal echocardiographic findings. On the other hand, postoperative right ventricular diastolic function was found to have improved significantly as the right ventricular E wave and E/A increased (9.5 ± 1.6 vs 7.6 ± 2.7 cm/s, *p* = 0.009 and 1.4 ± 0.2 vs 0.9 ± 0.2, *p* ≤ 0.01, respectively), while the A wave and isovolumic relaxation times (6.8 ± 2.1 vs 8.3 ± 3.4 cm/s, *p* < 0.03 and 55.2 ± 11.9 vs 87.2 ± 16.2 ms, *p* < 0.001, respectively) decreased. Although the S-wave peak amplitude decreased in group A patients, it did not reach statistical significance.

**Conclusions:**

Sequential, but not single, complete revascularisation of the right coronary artery appeared to improve the diastolic function of the right ventricle.

## Abstract

Coronary artery bypass graft surgery (CABG) is a well-known and proven method for myocardial revascularisation. The technique used (individual or sequential grafting) may be of importance with regard to long-term patency of these conduits.^1^ Saphenous vein grafts (SVGs) are still widely used but this is often limited to revascularisation of one tributary coronary vessel, and to situations in which a high starting flow is desirable immediately. On the other hand, use of sequential grafting of multiple coronary vessels in CABG surgery has been controversial since its introduction by Flemma^2^ and its amplification by Bartley,^3^ who suggested it to be the most extensive way of myocardial revascularisation.

The technical advantages of the sequential grafting method are the economical use of graft material and the decreased number of proximal anastomoses,^4^-^7^ as well as haemodynamic benefits.^5^,^8^,^9^ Although the effect of CABG on right ventricular (RV) function has been clearly demonstrated, the effect of sequential grafting of the right coronary artery remains to be clarified.

In this study we aimed to determine the effects of complete revascularisation of the right coronary artery (RCA) via sequential venous bypass grafting on the RV diastolic function as determined by tissue Doppler echocardiography.

## Methods

The study was approved by the local ethics committee and all subjects gave informed consent. Thirty-five (19 men and 16 women) consecutive patients undergoing CABG between 2006 and 2009 were enrolled into this prospective study. Group A (*n* = 20) included patients who had sequential right coronary artery bypass and group B (*n* = 15) included the remainder who could not have sequential bypass due to anatomical or technical reasons. All patients had significant coronary artery disease (CAD) and were accepted for CABG within two months of the diagnostic coronary angiography. Transthoracic echocardiography was performed before the CABG operation.

Patients who had isolated coronary artery disease not associated with any other cardiac pathology and who had a left ventricular ejection fraction > 50% were included in the study. In all patients, there were severe, diffuse and multiple atherosclerotic lesions on the right coronary artery. All the left anterior descending (LAD) lesions were located proximally.

Demographic data, including risk factors were recorded. Presence of hypertension was defined as systolic blood pressure > 140 mmHg and diastolic blood pressure > 90 mmHg on two or more occasions, or use of an antihypertensive drug. Patients who were on antidiabetic medication (insulin or oral hypoglycaemic) at the study entry or whose fasting blood glucose levels were higher than 125 mg/dl were considered to have diabetes mellitus. Hypercholesterolaemia was defined as total cholesterol level > 200 mg/dl or any medication being used. Patients were classified as cigarette smokers if they had smoked within the last 10 years. None of the patients had acute coronary syndrome.

Exclusion criteria included a history of recent right-side myocardial infarction, left ventricle systolic dysfunction (ejection fraction < 50%), right ventricular ejection fraction < 40%, presence of atrial fibrillation, previous CABG, significant valvular heart disease, pulmonary hypertension (> 50 mmHg), significant pulmonary disease, or left bundle branch block on electrocardiogram.

All echocardiographic measurements were performed by a single experienced investigator using an ultrasound imaging system (Vingmed 5, General Electric, USA) with a 2.5-MHz transducer equipped with pulsed-wave tissue Doppler. Heart rate during the examination was kept at 60 to 100 beats/min. Measurements were performed according to the recommendations of the American Society of Echocardiography.^10^ For M-mode, two-dimentional and Doppler examination, a 2.5-mHz transducer was used.

Pulmonary artery systolic pressure was calculated as the sum of the pressure gradient measured from the tricuspid regurgitation wave and estimated right atrial pressure.^11^ After the conventional echocardiographic examination, RV diastolic function was determined on the apical four-chamber view using a 2.5-mHz transducer.

With the subjects in the lateral supine position, tissue Doppler recordings were obtained from the standard parasternal and apical views. Three major velocities were recorded at the annular sites: the peak major positive systolic velocity when the annulus moved towards the apex, and two major negative velocities when the annulus moved back towards the base (one during the early phase of diastole and the other during the late phase of diastole). The velocities were recorded online at a sweep speed of 50 mm/s. A mean of five consecutive cycles was used for the calculations of all echo-Doppler parameters. RV ejection fraction was determined using the ellipsoidal shell method. All measurements were performed one month after the operation.

All operations were performed by the same surgeon who had 10 years of experience of coronary artery surgery. Complete revascularisation was the ultimate goal in all patients. A median sternotomy was performed in all patients. The *in situ* LIMA was always the graft of choice for revascularisation of the LAD. The LIMA was harvested with a pedicle and preserved in a sponge with papaverine to avoid spasm. SVGs were used for revascularisation of the remaining coronary vessels. The pericardium was opened, followed by general heparinisation, and aortic and venous cannulation using a double-stage atrial cannula, after which cardiopulmonary bypass was initiated.

**Fig. 1:**
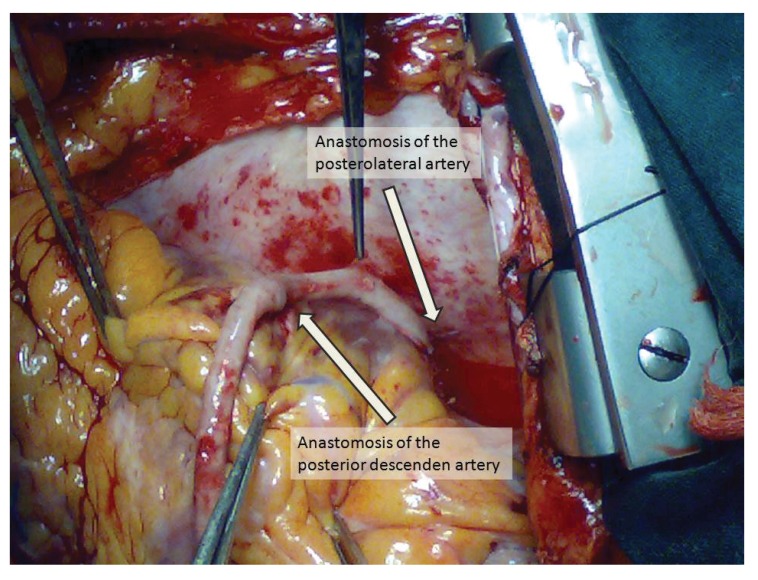
Intra-operative view of the right sequential bypass.

Cardiac arrest was maintained by warm blood cardioplegia under moderate hypothermia. A sequential venous graft was used for revascularisation of the RCA. The choice between individual and sequential technique was based on the anatomical position of the vessels and grafts. All coronary anastomoses were performed using a double-armed 7-0 polypropylene suture with a continuous suturing technique. Distal anastomosis was done end to side. Side-to-side anastomoses were performed in a diamond shape (graft axis perpendicular to coronary arteriotomy) and end-to side anastomoses parallel to the native coronary vessel axis (Fig. 1).

The length of the graft and the distance between segments to be anastomosed were determined before the initiation of cross-clamping while the size of the right ventricle was optimal, as an ‘empty’ right ventricle would lead to erroneous selection of an over- or undersized graft. Proximal anastomoses were constructed on the ascending aorta with continuous double-armed 6-0 polypropylene sutures using a side clamp during rewarming. Complete revascularisation using the internal thoracic artery for left anterior descending lesions and saphenous vein grafts for other coronary arteries was the goal in all operations.

## Statistical analysis

Statistical analysis was performed with SPSS software (SPSS Inc, Chicago, IL, USA). Clinical data were expressed as mean values ± standard deviation and in percentages unless stated otherwise. Categorical variables were assessed using the Chi-square test. Continuous variables were compared using the unpaired Student’s *t*-test or Mann-Whitney *U*-test. A *p*-value < 0.05 was considered statistically significant in all comparisons.

## Results

The basal clinical and echocardiographic parameters for the patients is shown in Table 1. There were no significant differences between the two groups regarding demographic variables and basal echocardiographic parameters. Mean age was 62.3 ± 4.0 years in group A and 64.7 ± 6.2 years in group B (*p* > 0.05). Maximum pulmonary systolic pressures did not differ between the two groups (34.3 ± 13.3 vs 32.9 ± 10.8 mmHg, *p* > 0.05). There were no mortalities or complications noted in the postoperative period. RV ejection fractions before and after the operation were not different.

**Table 1:** Basal Demographic And Echocardiographic Findings

Demographic characteristics	*Group A (n = 20)*	*Group B (n =15)*	p-*value*
Age (years)	62.3 ± 4.0	64.7 ± 6.2	NS
Male gender, *n* (%)	12 (60)	8 (53)	NS
Hypertension, *n* (%)	14 (70)	9 (60)	NS
Diabetes mellitus, *n* (%)	7 (35)	5 (33)	NS
Smoking, *n* (%)	9 (45)	8 (53)	NS
Hyperlipidaemia, *n* (%)	5 (25)	5 (33)	NS
Family history, *n* (%)	7 (35)	4 (27)	NS
Previous MI, *n* (%)	2 (10)	2 (13)	NS
Basal echocardiographic findings			
Left atrial diameter (cm)	4.3 ± 0.5	4.4 ± 0.7	NS
PASP (mmHg)	34.3 ± 13.3	32.9 ± 10.8	NS
LVEDD (cm)	4.41 ± 0.9	4.39 ± 1.1	NS
LVESD (cm)	2.9 ± 0.4	2.8 ± 0.6	NS
RV S-wave peak velocity (cm/s)	12.7 ± 1.8	12.1 ± 2.5	NS
RV E-wave velocity (cm/s)	8.1 ± 2.0	7.9 ± 2.2	NS
RV A-wave velocity (cm/s)	7.2 ± 2.4	7.0 ± 1.7	NS
RV E/A	1.2 ± 0.3	1.1 ± 0.4	NS
RV IVRT (ms)	69.2 ± 14.6	72.1 ± 11.8	NS

PASP: pulmonary artery systolic pressure, LVEDD: left ventricular end-diastolic diameter, LVESD: left ventricular end-systolic diameter, RV: right ventricular, IVRT: isovolumic relaxation time, NS: non-significant.

Postoperative RV diastolic parameters are presented in Table 2. In the sequential graft group (A), RV mean E-wave peak amplitude and E/A ratio were observed to significantly increase (9.5 ± 1.6 vs 7.6 ± 2.7 cm/s, *p* = 0.009 and 1.4 ± 0.2 vs 0.9 ± 0.2, *p* = 0.01, respectively), while RV mean A-wave peak amplitude and isovolumic relaxation times were found to be significantly decreased (6.8 ± 2.1 vs 8.3 ± 3.4 cm/s, *p* < 0.0001 and 55.2 ± 11.9 vs 87.2 ± 16.2 ms, *p* < 0.0001, respectively). Although the right ventricular S-wave peak amplitude decreased in group A, it did not reach statistical significance (9.1 ± 1.3 vs 9.3 ± 1.2 cm/s; *p* > 0.05). These findings have suggested that RV diastolic function improved in the sequential CABG group of patients.

**Table 2:** Postoperative Findings Of Right Ventricular Tissue Doppler Examination

	*Group A (n = 20)*	*Group B (n = 15)*	p-*value*
RV S-wave peak velocity (cm/s)	9.1 ± 1.3	9.3 ± 1.2	NS
RV E-wave velocity (cm/s)	9.5 ± 1.6	7.6 ± 2.7	0.009
RV A-wave velocity (cm/s)	6.8 ± 2.1	8.3 ± 3.4	0.03
RV E/A	1.4 ± 0.2	0.9 ± 0.2	0.01
RV IVRT (m)	55.2 ± 11.9	87.2 ± 16.2	< 0.0001

RV: right ventricular, IVRT: isovolumic relaxation time, NS: non-significant.

## Discussion

Although the echocardiographic assessment of left ventricular function is simple and well established, there is no gold standard for assessing right ventricular function by standard echocardiography since the evaluation is complicated by the complex anatomy of the chamber. In general practice, physicians rely largely on non-invasive imaging methods for assessment of right ventricular function and two-dimensional echocardiography is still the mainstay for its analysis. Recently, alternative techniques have been proposed such as tissue Doppler imaging techniques, three-dimensional echocardiography, magnetic resonance imaging, and even invasive assessment of pressure–volume loops.^12^

RV systole comprises a complex pattern of contractions of the RV myocardium along its long and short axes as well as rotation along its longitudinal axis. Despite these caveats, decreased RV function after CABG has clearly been demonstrated using the amplitude of tricuspid annular motion assessed by conventional M-mode or two-dimensional echocardiography immediately and six months after the operation.^7^,^8^ Subsequently, systolic and diastolic tricuspid annular velocities recorded by tissue Doppler imaging were shown to represent the respective systolic and diastolic functions of the right ventricle.^13^,^14^ However, possible beneficial effects of sequential venous grafting of the RCA on right ventricular diastolic function, which the present study sought to examine, have not been assessed.

The findings of our study showed that RV diastolic function significantly improved in the patients who underwent saphenous bypass grafting of the RCA using the sequential technique, whereas the systolic function did not improve. To our knowledge, this issue has not been studied before. Decreased RV function is known to occur after CABG operation; both RV filling and RV contraction are impaired after CABG.^15^-^17^ Cardiac surgery with cardiopulmonary bypass, cardioplegia, peri-operative myocardial ischaemia, aortic clamping and pericardial disruption/adhesions have all been proposed as the underlying reasons for this phenomenon.

While the E wave reflects RV relaxation in tissue Doppler examinations, the A wave is associated with atrial activity. The E/A ratio shows the passive elongatory ability of RV myocardial fibres, and a ratio < 1.0 refers to abnormal compliance.

On the other hand, the isovolumic relaxation time (IVRT) corresponds to the energy-dependent phase of the cardiac cycle. It was found to be significantly higher in the sequential graft group of patients, which may translate into improved compliance. These improvements may be due to haemodynamic advantages of sequential grafting of the RCA, as O’Neill *et al.*^9^ demonstrated higher flow velocity in the proximal segment of a sequential bypass graft than in individual grafts. The higher the flow velocity, the better preserved the perfusion and diastolic function of the right atrium and ventricle may be. This hypothesis could also be supported by the fact that sequential grafting enables the operation to be completed more quickly, thereby diminishing the period of total circulatory bypass and the level of oxidative stress on the right ventricle.

It has been demonstrated that peak systolic velocity (S wave) < 11.5 cm/s identifies the presence of RV dysfunction with a sensitivity and specificity of 90 and 85%, respectively.^18^ Tricuspid annulus velocities also decrease in the early and late (one-year) postoperative period.^16^,^17^ Therefore, similar decreases in S-wave peak velocities after CABG in both groups (both < 11.5 cm/s) may initially suggest that the sequential technique had no positive effect on RV systolic function. On the other hand, observation of similar decreases in S-wave velocities in both groups may probably be due to the non-specific harmful effects of surgery.

This study has some important limitations. The sample size was relatively small. Only one site of the tricuspid annulus was studied. In clinical practice, recording of other tricuspid annular sites might not be feasible in all patients. In addition, no method was included to assess the global RV function. In fact, there is no consensus regarding the echocardiographic assessment of global RV function aside from the gold-standard MRI. Two-dimensional echocardiography using the ellipsoidal shell model for right ventricular volumetry provides usable results for clinical practice, and it has been reported that it yielded data on right ventricular function comparable to those from MRI.^19^,^20^ On the other hand, a non-invasive perfusion study could have been utilised to assess the significance of the reduced tricuspid annular velocities.

## Conclusions

In general clinical practice, two-dimensional echocardiography can still be used to assess right ventricular dysfunction. Both systolic and diastolic RV functions are very important parameters in the peri- and postoperative period after CABG. Reducing right ventricular diastolic dysfunction via selecting the sequential, not individual, bypass technique for revascularisation of the RCA could help patients recover more rapidly and possibly improve their prognosis. Large-scale and randomised studies are still needed for assessment of the effect of this technique on long-term morbidity and survival.
